# Phytochemical Screening, Polyphenols Content, Antioxidant Power, and Antibacterial Activity of* Herniaria hirsuta* from Morocco

**DOI:** 10.1155/2018/7470384

**Published:** 2018-10-30

**Authors:** Kenza Ammor, Dalila Bousta, Sanae Jennan, Bahia Bennani, Abdellah Chaqroune, Fatima Mahjoubi

**Affiliations:** ^1^Laboratory of Materials Engineering and Environment, Sidi Mohammed Ben Abdelah University, BP 30003, Fez, Morocco; ^2^Laboratory of Neuroendocrinology, Nutritional and Climatic Environment, Sidi Mohammed Ben Abdelah University, BP 30003, Fez, Morocco; ^3^Laboratory of Human Pathology, Biomedicine and Environment, Medical School and Pharmacy, BP 3000, Fez, Morocco

## Abstract

The aim of this study is to investigate* in vitro* antioxidant and antibacterial activities of the aqueous and hydroethanolic extracts for aerial parts of* Herniaria hirsuta*. Extracts were screened for their possible antioxidant activities by three tests: DPPH free radical-scavenging, reducing power, and molybdenum systems. The screening of antibacterial activity of extracts was individually evaluated against sixteen bacteria species using a disc diffusion method. Flavonoids, total phenols, and tannins content were performed for both extracts. It shows higher content in the hydroethanolic extract. The hydroethanolic extract showed a significant antioxidant activity for the three methods studies to the aqueous extract, but nonsignificant results compared to the reference (BHT). However, both extracts have negative effect on the strains studies for the antibacterial activity.

## 1. Introduction

The genus of* Herniaria* contains several species which are widely distributed in Europe, Asia, and North Africa.* H. glabra*,* H. hirsuta*, and* H. fontanesii* are traditionally used in Moroccan folk medicine for the treatment of biliary dyskinesia and (uro)lithiasis or as a diuretic. In Europe,* Herniaria Herba*, which can contain* H. glabra* and* H. hirsuta*, is used as a urological drug [1–5].

Some phytochemical research on these species revealed the presence of saponins, flavonoids, and coumarins, while* H. fontanesii* was reported to contain saponins A–D [6–8], which are bidesmosidic triterpenoid saponins, two monodesmosidic derivatives of medicagenic acid.* Herniaria* saponins E and F were isolated from the aerial parts of* H. hirsuta* [4]. Also* H. glabra* contained several mono- and bidesmosidic medicagenic acid derivatives, named* Herniaria* saponins [1–7, 9–12]. Among others, herniarin and umbelliferon were identified as coumarins, quercetin, and isorhamnetin derivatives as flavonoids being present in* Herniaria* species [13–17].

In traditional Moroccan medicine, Herniaria is widely used as a diuretic and for the treatment of kidney diseases. In Algeria, the plant is used with the sabline against gravel and catarrh of the bladder. It is used as a preventive treatment against the formation of kidney stones and urinary sand [18–20].

According to Fournier [21],* Herniaria* has been known for different indications such as edema, bladder catarrh, urine retention, albuminuria, uremia, renal colic, diabetes, most kidneys and bladder diseases, bronchial catarrh, pulmonary phthisis, jaundice, leucorrhea, syphilis, skin diseases due to impurities in the blood, and weak eyesight associated with albuminuria or disorders uremic.

On the outside, the very foamy decoction is used to dress the indolent wounds and various ulcers.* Herniaria hirsuta *L. is also known as aseptic, lightly spasmolytic and very active for the treatment of inflammations of the urinary tract, kidney, and gall bladder [22, 23].

*H. hirsuta* infusion has proven efficacy against urolithiasis and cholelithiasis; the work of [1–3, 24] focuses on the phytochemical characterization of this infusion using semipreparative HPLC, mass spectrometry, and NMR. Finally, aiming at the development of a standardized extract, this can be used in the prophylaxis treatment of stone diseases.

All these bioactive molecules highlighted also contribute to the virtues of the plant, considering in particular the benefits of polyphenols with respect to cardiovascular diseases [25] and the role of tannins and mucilage in the treatment of diabetes mellitus by their capacity to delay the release of glucose and their antioxidant effects [26, 27].

*H. hirsuta *belongs to Caryophyllaceae family which is not enough examined for antibacterial components regarding isolation and estimation of specific substances. However, antibacterial properties of* Caryophyllus aromaticus, *which is a member of the same family, are well known and characterized. Eugenol and isoeugenol are two main phenolic compounds which are responsible for most of antibacterial activity [28].

## 2. Material and Methods

### 2.1. Vegetal Material

In July 2015 we collected samples of aerial parts of* H. hirsuta* from the Middle Atlas Mountain in Morocco; the specimen identified was deposited in the Herbarium of Biotechnology and preservation of natural resources laboratory (BPNR), Sidi Mohammed Ben Abdellah University, Fez, Morocco.

### 2.2. Preparation of the Plant Extract

#### 2.2.1. Decoction

The extraction method was performed by taking 25g of dried and pulverized aerial part from* H. hirsuta* with 250 mL of distilled water. The mixture was heated (reflux system) for 30 minutes and then filtered using Whatman filter paper and concentrated under reduced pressure.

#### 2.2.2. Soxhlet Extraction

In a Soxhlet system, 25g of dried plant powder was put in a cellulose cartridge and extracted with 250 mL of ethanol-water (70/30). The extraction process continued until the solvent in siphon tube of an extractor became colorless. After that the extract was filtered and concentrated under reduced pressure.

### 2.3. Qualitative Phytochemical Analysis

Phytochemical screening of dry extracts was achieved through simple methods as described in [29–32].

### 2.4. Determination of the Total Phenolic Content

To determine the total phenolic content, we used the method of Folin-Ciocalteu [33]; we picked up 20*μ*L from each extract and mixed with 1.16mL of distilled water, 100 *μ*L of Folin-Ciocalteu reagent, and 300*μ*L of sodium carbonate (Na_2_ CO_3_) to 20%. The whole is incubated at 40°C for 30 minutes and the reading is taken against a white using a spectrophotometer at 760nm.

The absorbance was measured with a white made from distilled water using a spectrophotometer UV-Visible kind Selecta. A calibration curve was plotted for different concentrations of gallic acid. The data are presented as the average of triplicate analyses.

### 2.5. Determination of Flavonoid Contents

The flavonoids assay was performed according to the method described by [34]. A quantity of 0.5mL of the extract was mixed with 0.5 mL of aluminum chloride (AlCl_3_). After 1-hour incubation at room temperature, the absorbance is measured at 420nm.

Quantification of flavonoids is determined based on a linear calibration curve produced by the quercetin at different concentrations and under the same conditions as the sample. The results are expressed in microgram equivalents of quercetin per milligram extract (QE *μ*g/mg of extract). The data are presented as the average of triplicate analyses.

### 2.6. Determination of Tannins Contents

Defining tannins content requires 0.1mL of crude extracts put into tubes covered with aluminum foil. We added three milliliters of 4% vanillin (w/v) in methanol [35] and agitated the tubes with a mixer. Immediately after that 1.5 mL of concentrated HC1 was pipetted and the tubes were shaken again. The absorbance is read at 500 nm after being left at room temperature for 20 minutes. The results were plotted after gallic acid standard made in the same manner. The interference background of the crude extract was corrected by preparing the test without vanillin.

### 2.7. Evaluation of the Antioxidant Activity

#### 2.7.1. The DPPH Method

The free radical-scavenging activity of extracts was evaluated by 1,1-diphenyl-2-picrylhydrazyl radical (DPPH) according to the method reported by [36]. 50 *μ*L of different concentrations of the extracts was mixed with 2 ml of a 60 *μ*M DPPH methanolic solution; then we agitated the mixture vigorously and incubated it for 20 minutes in the dark at 25°C. The absorbance of the samples was measured by spectrophotometry at 517 nm. Butylated hydroxytoluene (BHT) was used as a positive control. The antiradical capacity of the extracts studied was calculated using the following formula:

(1) $$\begin{array}{c} \text{DPPH scavenging effect }\left(\;\%\;\right)=\frac{\left[\left(A_{0}-A_{1}\right)\right]}{A_{0}}\times 100 \end{array}$$

where A_0_ is the absorbance of the control and A_1_ is the absorbance of the sample.

The antiradical activity is expressed as IC_50_, which is the antiradical concentration required to cause 50% of inhibition. A lower IC_50_ value corresponds to a higher antioxidant capacity of the extract.

The IC_50_ is calculated by plotting inhibition percentages against concentrations of the sample. The experiment was repeated three times and the results were expressed as mean ± SD.

#### 2.7.2. Ferric-Reducing Antioxidant Power (FRAP)

The reductive potential is examined by transforming Fe^+3^ to Fe^+2^ in the presence of an antioxidant; this test is realized according to the procedure of Oyaizu [37]. In a test tube we added 1 mL of sample solution, 2.5 mL of phosphate buffer (0.2 M, pH 6.6), and 2.5 mL of potassium ferricyanide [K_3_Fe (CN)_6_] (1%). The mixture should be incubated at 50°C for 20 min. After incubation, 2.5 mL of trichloroacetic acid 10% is added to the mixture and centrifuged at 3000 rpm for 10 min. Then, 2.5 mL of the supernatant was mixed with 2.5 mL of distilled water and 0.5 mL of FeCl_3_ (0.1%) and the absorbance was measured spectrophotometrically at 700 nm.

#### 2.7.3. Evaluation of the Total Antioxidant Capacity by Phosphomolybdenum Method

Total antioxidant capacity (TAC) of the extracts was evaluated by phosphomolybdenum method Prieto [38]. This technique is based on the reduction of molybdenum Mo (VI) to Mo (V) by the extract and subsequent formation of a green phosphate/Mo (V) complex at acid pH. A volume of 0.3 mL of the extract was mixed with 3 mL of the reagent solution (0.6 M sulfuric acid, 28 mM sodium, and 4 mM of ammonium molybdate). The tubes were incubated at 95°C for 90 min. After cooling, the absorbance of the solutions is measured at 695 nm against the blank which contains 3 mL of the reagent solution and 0.3 mL of methanol and which was incubated under the same conditions as the sample.

Total antioxidant capacity is expressed in milligrams of ascorbic acid per gram of dry matter (mg EAA / g MS). The experiments are repeated three times.

### 2.8. Bacterial Inhibitory Effect by the Disc Diffusion Method

The inhibition of bacterial growth* in vitro* was studied using disc diffusion method. Filter Discs (6 mm in diameter) were sterilized and imbibed with various solutions of 0.1 g/mL at a rate of 10*μ*L and deposited on the surface of a solid medium seeded with bacterial suspensions.

The tested species were* E. coli*,* Staphylococcus aureus*,* Klebsiella pneumoniae*,* Pseudomonas aeruginosa*,* Proteus mirabilis*,* Staphylococcus saprophyticus*,* Serratia*,* Citrobacter*,* Enterococcus faecalis*,* Ralstonia pickettii*,* Aeromonas hydrophila*,* Enterobacter cloacae*,* Chryseobacterium indologenes*, and* Acinetobacter baumannii*. The incubation times and temperatures were 24 H at 37°C. The antimicrobial activity was determined by the measurement of the inhibition zone around the impregnated disc. Each experiment was performed in triplicate and the mean value was taken as diameter of inhibition. Disks soaked in water or ethanol were used as a negative control [39].

## 3. Results and Discussion

### 3.1. Extraction Yield

The extraction of* H. hirsuta* aerial parts showed a higher yield for the hydroethanolic extract with a value of 31.20%, followed by the aqueous extract with a yield of 28.73%.

### 3.2. Phytochemical Screening

The phytochemical tests carried out on the aqueous and hydroethanolic extracts of the aerial parts of* H. hirsuta* show the presence of gallic tannins, flavonoids, and coumarins in both extracts (Table 1). The mucilages are present only in the aqueous extract of the plant. Alkaloids, triterpene, sterols, heterosids, and triterpenes are present only in the hydroethanolic extract. The cardiac glicosides, oses, and holosides are absent in both extracts.

### 3.3. Total Phenolic, Flavonoid, and Tannins Contents

The total phenol content was determined in comparison with a standard which is gallic acid. The results were expressed in terms of mg GA/g of extract (Table 2). The hydroalcoholic extract shows better polyphenol content than the aqueous extract.

(2) $$\begin{array}{c} Y=1.575X-0.0229\;\left(R^{2}=0.959\right). \end{array}$$

The total flavonoid content was determined in comparison with quercetin. The hydroalcoholic extract contains more flavonoid content than the aqueous extract.

(3) $$\begin{array}{c} Y=0.0489x-0.0416\;\left(R^{2}=0.977\right). \end{array}$$

The tannins content was determined in comparison with gallic acid. The hydroalcoholic extract has higher flavonoid content than the aqueous extract.

(4) $$\begin{array}{c} Y=\text{0,2482}X+0.1758\;\left(R^{2}=\text{0,9235}\right). \end{array}$$

### 3.4. Antioxidant Activity

#### 3.4.1. Determination of Free Radical Scavenging Activity by DPPH Method

The anti-free radical capacity of the hydroalcoholic and aqueous extracts of* H. hirsuta* was tested by the DPPH method using BHT as a reference standard. The concentration varies between 0.01 and 6 mg/mL.

Inhibition zero was considered for the solution that contained only DPPH without plant extract. The result showed an antioxidant activity for the two extracts with an IC_50_ = 4.03 mg/mL for the hydroethanolic extract and an IC_50_ = 6.67 mg/mL for the aqueous extract compared to BHT, the standard which has an IC_50_ = 0.17 mg/mL (Table 3).

#### 3.4.2. Reducing Power by FRAP Method

Potential measures reduce the ability of a sample to act as an electron donor and, therefore, react with a radical connection by converting it to more stable products and thus end radical chain reactions.

**Figure 1:**
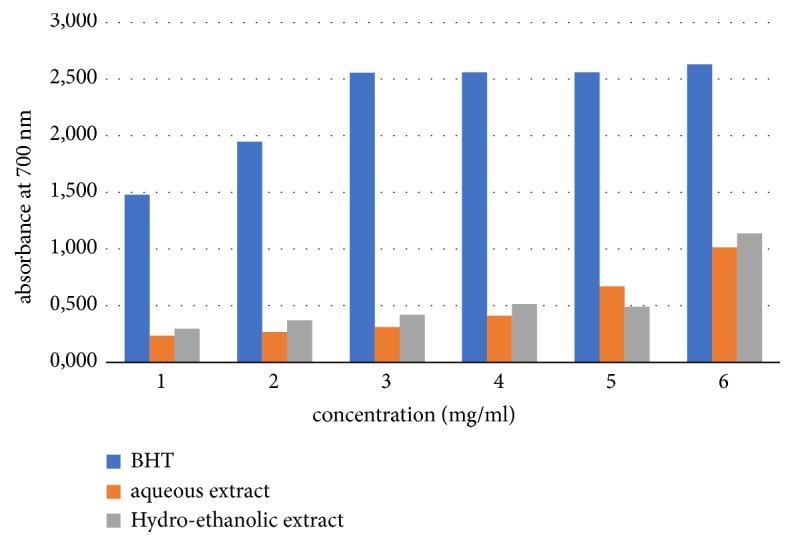
Reducing power of different* H. hirsuta *extracts compared to BHT standard.

It is shown, in Figure 1, that the power of reduction of the extracts increases with the concentration. The hydroethanolic extract showed stronger reduction of power compared to the aqueous extract.

#### 3.4.3. Evaluation of the Total Antioxidant Capacity by Phosphomolybdenum Method

The total antioxidant activity of the aqueous and hydroalcoholic extracts is expressed in equivalents of BHT (Table 4). The test is based on the reduction of Mo (VI) to Mo (V) by the extract and the subsequent formation of a phosphate of a complex green/Mo (V) complex at an acidic pH. The method is quantitative phosphomolybdenum since the antioxidant activity is expressed in the number of equivalents of BHT. The hydroethanolic extract has higher antioxidant capacity than that of the aqueous extract.

### 3.5. Antibacterial Activity

The antibacterial activities of* Herniaria hirsuta* extracts were tested* in vitro *against sixteen pathogenic bacteria using the disc agar diffusion technique. The antibacterial activity of these extracts was qualitatively and quantitatively assessed by the presence or absence of inhibition zones and zone diameter measures, respectively. Control treatment (alcohol 20%) did not show an inhibitory effect on any of the bacteria.  Table 5 summarizes the microbial growth inhibition by each extract.

According to the results,* H. hirsuta *extracts show similar activity against tested species. They showed hardly any activity against* Aeromonas hydrophila* and* Enterobacter cloacae,* while they showed no activity against the rest of species.

## 4. Discussion

Concerning our study, the phytochemical screening of the aerial parts of* Herniaria hirsuta* was realized on the aqueous and hydroalcoholic extracts. The results revealed the presence of gallic tannin, flavonoids, and coumarins in both extracts. The mucilages are present only in the aqueous extract. Alkaloids, triterperne, sterols, heterosides, and triterpenes are present only in the hydroalcoholic extract. On the other hand, the cardiac glicosides, oses, and holosides are absent in both extracts. Some phytochemical research on these species revealed the presence of flavonoids and coumarins [7]. Among others, herniarin and umbelliferon were identified as coumarins and quercetin and isorhamnetin derivatives as flavonoids being present in* Herniaria* species [13–17].

The flavonoids of* Herniaria* species, which have a widespread distribution in the Mediterranean area, have been until now little studied [15, 40]. In a previous investigation of the secondary metabolites of* Herniaria fontanesii* [15] isolated a triterpenoid and reported the characterization of a new flavonoid derivative from the aerial parts of the same plant, in addition to two known flavonoids.

The hydroalcoholic extract has higher values of phenols content than the aqueous extract. Comparing our results to those found by [41] the ethanolic extract has a value of 28.2 mg of GA/g of extract contrariwise; we found 71,39 mg of GA/g of extract for the hydroalcoholic extract. For the aqueous extract, we found a value of 56.57 mg of GA/g of extract which is double their results. Regarding the flavonoids content we found 13.93 and 7.72 mg of QE/g of hydroalcoholic and aqueous extracts, compared to their results, consecutively 4.6 and 3.7 mg of QE/g of extract. Concerning the tannins content our results are lower than they found; we got a result of 2.37 and 2.28 mg of GA/g of hydroalcoholic and aqueous extracts but 12.1 and 8.1 mg of GA/g of extract for their finding. Several studies have shown that hydroalcoholic extracts contain more compounds in tannins, phenols, and flavonoids than aqueous extracts [42].

Concerning the antioxidant activity of* Herniaria hirsuta* there are no previous studies achieved. In the present study, the antioxidant capacity of* Herniaria hirsuta *extracts was evaluated using three tests used for the characterization of plant extracts: DPPH, phosphomolybdenum, and the reducing power. In our study we conclude that the hydroethanolic extract exhibited a higher antioxidant activity than the aqueous extract. There are higher amounts of all examined compounds in hydroethanolic than in aqueous extract which is in a direct relation to its superior antibacterial property.

In our study, extracts of* Herniaria hirsuta* show similar activity against tested species. They showed hardly any activity against* Aeromonas hydrophila* and* Enterobacter cloacae.* So, we can conclude that both extracts are devoid of any effect on the sixteen strains studied, the same results as [39]. Antibacterial effect against* E. coli *strain was demonstrated* in vitro*, using agar, the dilution method [43]; the ethanol extract produced significantly higher values of inhibition zones than aqueous extract.

These results suggest that* H. hirsuta* could be used as source of diverse bioactive compounds. It is then necessary to identify and isolate the compounds that are responsible for the antioxidant properties.

## Figures and Tables

**Table 1:** Phytochemical screening of the aqueous and the hydroethanolic extracts of *H. hirsuta*.

Chemical constituent			Hydro ethanolic Extract	aqueous Extract
polyphenols	Tannins	Total Tannins	+	+
		Gallic tannins	+	+
		catechin tannins	-	-
	Flavonoids	Flavonols	+	+
		Anthocyanes	-	-
		Leucoanthocyanes	-	-

Alcaloids			+	-

triterpenes et sterol			+	-

Mucilage			-	+

Saponosids			-	-

heteroside steroidic			-	-

triterpenes heterosids			+	-

Coumaines			+	+

Glycosides cardiaque			-	-

oses and holosides			-	-

+: presence; -: absence.

**Table 2:** Total phenolic, flavonoids, and tannins contents in the aqueous and hydroethanolic extracts of *H. hirsuta.*

Extract	Total phenolic content (mg GAE/g extract)	Flavonoids content (mg QE/g extract)	Tannins content (mg GAE/g extract)
Hydro ethanolic	71,39±1,13	13,93±0,12	2,37 ±0,08

Aqueous	56,57±6,77	7,72±0,164	2,28±0,05

**Table 3:** Inhibition activity of BHT and different extracts of *H. hirsuta.*

Extract	DPPH IC_50_ (mg/ml)
Hydro-ethanolic	4.03
aqueous	6.67
BHT	0,17

**Table 4:** Total antioxidant activity of *H. hirsuta* extracts.

	TPC (mg BHT/g of extract)

Hydro alcoholic extract	44,94±4,49

Aqueous extract	26,13±2,16

**Table 5:** Antibacterial activity expressed and inhibition zone diameters (mm) of *H. hirsuta *extracts by the disc diffusion method.

		Inhibition diameters (cm)	
	strains	*H. hirsuta* hydro alcoholic extract	*H. hirsuta* aqueous extract
gram (-)	*Chryseobacterium indologenes*	-	-
	*Citrobacter*	-	-
	*Aeromonas hydrophila*	8	8
	*Enterobacter cloacae*	8	9
	*Serratia*	-	-
	*Ralstonia pickettii*	-	-
	*Pseudomonas aeruginosa*	-	-
	*Escherichia coli*	-	-
	*Proteus mirabilis*	-	-
	*Acinetobacter*	-	-
	*Acinetobacter baumannii*	-	-
	*Pseudomonas*	-	-
	*Klebsiella*	-	-

gram (+)	*Enterococcus faecalis*	-	-
	*Staphylococcus aureus*	-	-
	*Staphylococcus aureus*	-	-

+: presence; -: absence.

## Data Availability

These data are included in the article; they represent an average analysis of three repetitions and are present in tables with their standard deviation.
